# Characteristics of the phenotype of mixed cardiomyopathy in patients with implantable cardioverter-defibrillators

**DOI:** 10.1007/s10840-023-01577-x

**Published:** 2023-06-05

**Authors:** Deep Chandh Raja, Indira Samarawickrema, Sarat Krishna Menon, Rikvin Singh, Abhinav Mehta, Lukah Q. Tuan, Ulhas Pandurangi, Sanjiv Jain, David J. Callans, Francis E. Marchlinski, Walter P. Abhayaratna, Prashanthan Sanders, Rajeev K. Pathak

**Affiliations:** 1 ANU School of Medicine and Psychology, Australian National University, 54 Mills Road, Australian Capital Territory Acton, 2601 Australia; 2 Canberra Heart Rhythm, Suite 14, 2 Garran Place, Australian Capital Territory Garran, 2605 Australia; 3 Cardiac Electrophysiology Unit, Department of Cardiology, Canberra Health Services, Yamba Drive, Australian Capital Territory Garran, Australia; 4 University of Canberra, Canberra, Australian Capital Territory Australia; 5 University of Newcastle, Newcastle, NSW Australia; 6 Madras Medial Mission, Chennai, India; 7 Electrophysiology Section, Hospital of the University of Pennsylvania, Philadelphia, PA USA; 8 Centre for Heart Rhythm Disorders, University of Adelaide and Royal Adelaide Hospital, Adelaide, Australia

**Keywords:** Ischemic cardiomyopathy, Nonischemic cardiomyopathy, Mixed cardiomyopathy, Implantable-cardioverter defibrillator, Device shocks, Mortality

## Abstract

**Background or Purpose:**

The prognosis of m ixed cardiomyopathy (CMP) in patients with implanted cardioverter-defibrillators (ICDs) has not been investigated. We aim to study the demographic, clinical, device therapies and survival characteristics of mixed CMP in a cohort of patients implanted with a defibrillator.

**Methods:**

The term mixed CMP was used to categorise patients with impaired left ventricular ejection fraction attributed to documented non-ischemic triggers with concomitant moderate coronary artery disease. This is a single center observational cohort of 526 patients with a mean follow-up of 8.7 ± 3.5 years.

**Results:**

There were 42.5% patients with ischemic cardiomyopathy (ICM), 26.9% with non-ischemic cardiomyopathy (NICM) and 30.6% with mixed CMP. Mixed CMP, compared to NICM, was associated with higher mean age (69.1 ± 9.6 years), atrial fibrillation (55.3%) and greater incidence of comorbidities. The proportion of patients with mixed CMP receiving device shocks was 23.6%, compared to 18.4% in NICM and 27% in ICM. The VT cycle length recorded in mixed CMP (281.6 ± 43.1 ms) was comparable with ICM (282.5 ± 44 ms; *p* = 0.9) and lesser than NICM (297.7 ± 48.7 ms; *p* = 0.1). All-cause mortality in mixed CMP (21.1%) was similar to ICM (20.1%; *p* = 0.8) and higher than NICM (15.6%; *p* = 0.2). The Kaplan–Meier curves revealed hazards of 1.57 (95% CI: 0.91, 2.68) for mixed CMP compared to NICM.

**Conclusion:**

In a cohort of patients with ICD, the group with mixed CMP represents a phenotype predominantly comprised of the elderly with a higher incidence of comorbidities. Mixed CMP resembles ICM in terms of number of device shocks and VT cycle length. Trends of long-term prognosis of patients with mixed CMP are worse than NICM and similar to ICM.

**Supplementary Information:**

The online version contains supplementary material available at 10.1007/s10840-023-01577-x.

## Introduction

Significant progress has been made with the tools in diagnosis and management of heart failure. One of these advances is the prevention of sudden cardiac death (SCD) with implantable cardioverter-defibrillators (ICD) [1]. Regardless, the long-term mortality rates in heart failure patients, even with ICD, continue to remain as high as 50% at 10 years [2, 3]. These trends are worse in ischemic (ICM) than in nonischemic (NICM) forms of cardiomyopathy (CMP) [4]. It may not be right to simplify the burden of coronary artery disease (CAD) in patients with cardiomyopathies as a binary component of epicardial stenosis of more than or less than 75%, and thus attribute the heart failure to ischemic or nonischemic aetiologies [5]. There is ongoing research on ways to detect ischemia in cardiomyopathies [6]. The studies on the prognosis of concomitant CAD in dilated cardiomyopathies (DCM) are few, and these studies have reported the prognosis of the association of CAD in only idiopathic DCM [7-9]. Thus, the effect of moderate CAD coexisting with DCM with definite non-ischemic triggers is largely unexplored. The resultant phenotype of ‘mixed cardiomyopathy’ might identify clinical and outcome characteristics that are distinct from ICM or NICM and may impact on clinical management. This phenotype is gaining attention of late and the prognosis in terms of increased ventricular arrhythmia burden seems to parallel ICM [10, 11]. We aim to study the demographic, clinical, device therapies and survival characteristics of mixed CMP in a cohort of patients implanted with a defibrillator.

## Methods

The Canberra Hospital (TCH) device registry is a prospectively maintained database of implanted cardiac devices. The demographic and clinical data is being recorded at scheduled clinic visits and the device data is being interrogated through scheduled or unscheduled clinic visits and remote monitoring of the devices. In this study, consecutive patients receiving an ICD between January 2005 and June 2019 who had regular interrogation (clinical or remote transmission) of the implanted ICD in the follow-up and with an invasive coronary angiogram to rule out coronary artery disease were included. The identity of these patients was linked with the National Death Index (NDI) obtained from the Australian Institute of Health and Welfare (AIHW) to confirm the survival status and cause of death. The following patients were excluded from the study: incomplete clinical or device data; no survival data; in-hospital or immediate post-procedure (< 30 days) deaths; channelopathies.

The study complies with the Declaration of Helsinki and was approved by the Human Research Ethics Committee (2019/LRE/0127) and the AIHW Ethics Committee (EO2020/1/1102). The primary objective of the study was to analyse the characteristics of the demographic variables, clinical variables, device therapies and survival data of patients receiving an ICD in patients with mixed CMP in comparison with ICM and NICM. The secondary objectives were to analyse the characteristics of clinical, device therapies and mortality in non-survivors in the total cohort and to identify the significant predictors of mortality in the total cohort.

### Data collection

Demographic and clinical variables including history of diabetes mellitus, hypertension, chronic kidney disease (CKD), lung disease, malignancy, alcohol/ drug abuse, renal functions and echocardiographic findings including type and severity of valve pathologies were recorded. The left ventricular ejection fraction (LVEF) at implant and at the last follow-up was recorded. History of CAD, myocardial infarction (MI), percutaneous coronary intervention (PCI) history of bypass surgery, valve replacement; documented atrial and ventricular arrhythmias; list of anti-arrhythmic and heart failure medications; symptoms of syncope or sudden cardiac arrest (SCA); history of radiofrequency ablation (RFA) for VT in relation to the time of the ICD implant was collected. The following device characteristics were collected: information on clinical interrogation during a scheduled clinic visit or remote transmission, type of ICD, the programming zones of the ICD, date of first and second therapy from the device, verification of the type of tachyarrhythmia and the type of therapies delivered verified with the stored intracardiac electrograms (EGMs), change in the programming parameters, ventricular tachyarrhythmia (VT) storms, minimum cycle length of the recorded VT (1st and 2nd episode was taken into account), date and number of generator changes, therapies after generator change. The survival characteristics were collected from the NDI.

### Study definitions

1. Coronary artery disease (CAD) was defined by the presence of stenosis $\ge$ 50% in atleast one of three major epicardial vessels or $\ge$ 30% in the left main vessel. Lesions on coronary angiography (CAG) were graded visually by two cardiologists on the following ordinal scale: 0 to < 50%, $\ge$ 50 to < 75, $\ge$ 75% and 100%. The interobserver agreement for both grading of stenosis and location of CAD was calculated. The final consensus was reached upon by mutual agreement.
2. ICM was defined as those patients with impaired LVEF in whom there was a history of MI, evidence of prior MI in form of q-waves in ECG or regional wall motion abnormalities in echocardiogram or $\ge$ 75% coronary artery stenosis in one of the major epicardial vessels or $\ge$ 50% coronary artery stenosis in the left main coronary artery as evidenced in a diagnostic coronary angiogram (CAG) [12].
3. NICM was defined as those patients with depressed LV systolic function (< 50%) in whom moderate to severe CAD ($\ge$ 50% stenosis in one of the epicardial coronary vessels) was ruled out by a CAG and with no history suggestive of MI. After corroborative evidence from electrocardiography, echocardiography, cardiac MRI, PET scan and genomic assessment, the aetiopathogenesis of NICM was assigned and included the following; post-myocarditis sequelae, arrhythmogenic right ventricular cardiomyopathy, sarcoidosis, hypertrophic cardiomyopathy, infiltrative cardiomyopathy (amyloidosis, hemochromatosis), non-compaction (dilated and low LVEF associated with features of non-compaction documented by echocardiogram or cardiac MRI), valvular heart disease (severe valvular stenosis/ regurgitation leading to dilatation of heart and low LVEF), alcohol-related (documented alcohol abuse or dependence leading to deterioration in LVEF), congenital heart disease (including post-operative patients with persisting heart defects or new onset valvular diseases), tachy-cardiomyopathy, and chemotherapy-related cardiomyopathy. Patients with no known aetiology other than those stated above, but with LVEF ≤ 35% were classified as idiopathic dilated cardiomyopathy (DCM).
4. The term mixed CMP was used in this study to categorise patients with depressed LV systolic function (< 50%), a documented non-ischemic aetiology and with moderate CAD ($\ge$ 50% and < 75% stenosis) in one or more of left anterior descending artery (LAD), left circumflex artery (LCX), right coronary artery (RCA), or 30–50% stenosis involving the left main coronary artery.
5. Minimum cycle length of VT was calculated based on the least measured near-field EGM intervals in the available intracardiac traces. The average of first 10 intervals was considered in case of unstable intervals. Additional study definitions are incorporated in the supplement.

### Statistical analysis

Categorical variables are summarised as percentages. Normally distributed continuous data is expressed as mean ± SD, and non-normally distributed data is expressed as median with interquartile range of 25th and 70th percentiles. For comparing variables, we used a *χ*^*2*^-test (categorical variables), a t-test (normally distributed continuous variables) and a Mann–Whitney *U* test (non-normal continuous variables). The kappa statistics were used to calculate the inter-observer variability in the extent and location of CAD detected in the coronary angiograms. Cumulative hazard and the survival curves following ICD intervention were analysed with the Kaplan–Meier survival analysis method and the statistical comparison using the log-rank test. The Cox proportional hazards regression models were used to determine the predictors of survival. The coefficients were expressed as hazard ratios with 95% confidence intervals. *p* value < 0.05 was considered statistically significant.

## Results

**Fig. 1:**
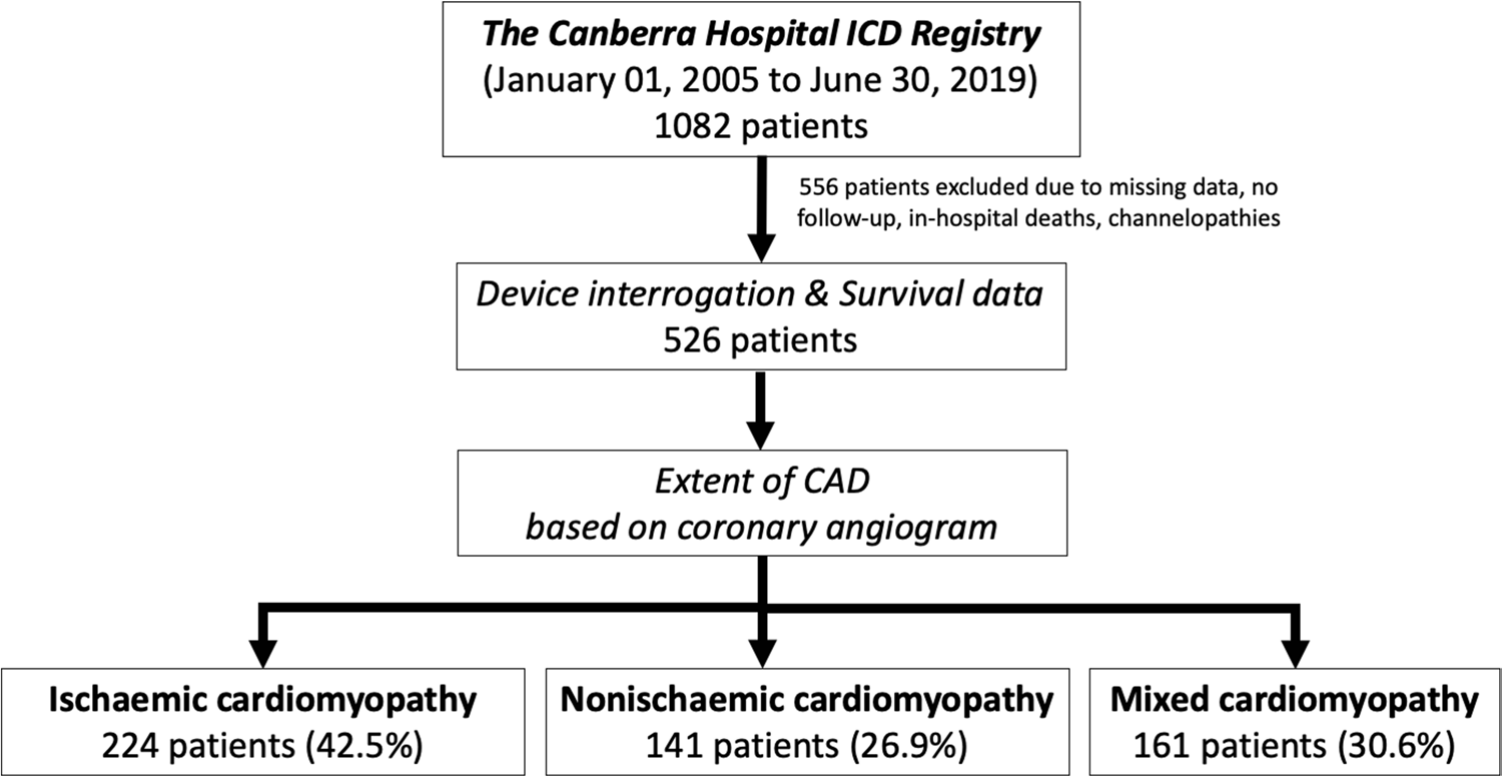
Is the flow diagram illustrating the selection of the patients from the ICD registry and grouping of the cohort into the three forms of cardiomyopathy

In this study, 526 patients were followed up for a mean period of 8.7 ± 3.5 years. The total cohort comprised of 224 patients of ICM (42.5%), 141 patients of NICM (26.9%) and 161 patients of mixed CMP (30.6%) (Fig. 1).

### Demographic and clinical characteristics (Table 1)

**Table 1:** Clinical and device therapy characteristics in the three groups of cardiomyopathies in patients with ICD implant

Variables	Total (*n* = 526)	ICM (*n* = 224)	NICM (*n* = 141)	Mixed CMP (*n* = 161)
Age at implant (years)	64 ± 13	66.3 ± 10.9 *	54.4 ± 14.5	69.1 ± 9.6^+, $^
Men	432 (82.1)	206 (92)*	94 (66.7)	132 (82)^+, $^
Diabetes mellitus	173 (33)	100 (44.8)*	19 (13.5)	54 (33.5)^+, $^
Hypertension	290 (55.1)	139 (62.1)*	51 (36.2)	100 (62.1)^$^
Chronic lung diseases	45 (8.6)	21 (9.4)*	3 (2.1)	21 (13)^$^
Chronic kidney disease	91 (17.3)	45 (20.1)*	10 (7.1)	36 (22.4)^$^
Alcohol abuse	60 (11.4)	0 (0)*	24 (17)	36 (22.4)^+^
Malignancy	71 (13.5)	6 (2.7)*	16 (11.3)	49 (30.4)^+, $^
Atrial fibrillation	196 (37.3)	64 (28.6)	43 (30.5)	89 (55.3)^+, $^
Left ventricle ejection fraction (in %)	35 ± 10.9	32.7 ± 8.3*	40.9 ± 14.2	32.9 ± 8.6^$^
Estimated glomerular filtration rate	81.7 ± 26.8	79 ± 29*	89 ± 25.7	79.2 ± 23.4^$^
Coronary artery disease (≥ 50% stenosis)	385 (73.2)	224 (100)*	0 (0)	161 (100)^$^
Percutaneous coronary intervention	117 (30.7)	117 (53.2)*	0 (0)	0 (0)^+^
Coronary artery bypass surgery	100 (26)	100 (44.8)*	0 (0)	0 (0)^+^
Syncope	119 (22.6)	39 (17.4)*	45 (31.9)	35 (21.7)
Cardiac arrest	107 (20.3)	46 (20.5)	23 (16.3)	38 (23.6)
NYHA class 2	282 (53.6)	150 (67)*	56 (39.7)	76 (47.2)^+, $^
NYHA class 3	136 (25.9)	50 (22.3)*	33 (23.4)	53 (32.9)
Secondary prevention	251 (47.7)	126 (56.3)*	54 (38.3)	71 (44.1)^+^
Betablocker usage	504 (96.4)	215 (96)	133 (95.7)	156 (97.5)
Amiodarone usage	169 (32.3)	85 (37.9)	39 (28.1)	45 (28.1)
ACEi-ARB usage	383 (73.2)	174 (77.7)*	78 (56.1)	131 (81.9)^$^
Cardiac resynchronisation therapy	121 (23)	48 (21.4)	26 (18.4)	47 (29.2)^$^
Minimum VT cycle length (milliseconds)	286.8 ± 45.6	282.5 ± 44	297.7 ± 48.7	281.6 ± 43.1
Therapies received	184 (35.7)	88 (41.1)*	41 (29.1)	55 (34.2)
Shocks received	125 (23.7)	61 (27.2)	26 (18.4)	38 (23.6)
Appropriate shocks	86 (16.3)	42 (18.8)	17 (12.1)	27 (16.7)
Inappropriate shocks	39 (7.4)	19 (8.4)	9 (6.4)	11 (6.8)
Median number of therapies	5 (2, 15.8)	4.5 (1, 12)	4 (2, 16)	8 (2, 27)
VT storms	44 (8.3)	18 (8)	14 (9.9)	12 (7.4)
Generator change	96 (18.3)	40 (17.9)	31 (22)	25 (15.5)
Therapies post generator change	34 (34.7)	16 (39)	9 (29)	9 (34.6)
Time-to-first therapy (years)	2.4 ± 2.8	2.4 ± 2.7	2.8 ± 3.4	2 ± 2.3
Time-to first appropriate shock (years)	2.3 ± 2.8	2.4 ± 2.9	2.6 ± 3.5	1.8 ± 1.9

*ICD* implantable cardioverter-defibrillator, *ICM* ischemic cardiomyopathy, *NICM* nonischemic cardiomyopathy, *Mixed CMP* mixed cardiomyopathy, *NYHA* New York Heart Association classification, *VT* Ventricular tachycardia

*P* values < 0.05 have been denoted as * for the significant differences between ICM and NICM groups, + for the significant differences between mixed CMP and ICM groups, $ for the significant differences between Mixed CMP and NICM groups

Categorical variables have been presented as frequencies (proportions in %), continuous variables have been presented as mean ± standard deviation with 95% confidence intervals, medians have been presented as average (25th, 70th percentiles)

The mean age of patients with mixed CMP (69.1 ± 9.6 years) was higher compared to both ICM (66.3 ± 10.9 years; *p* = 0.008) and NICM (54.4 ± 14.5 years; *p* < 0.001). The mean LVEF in patients with mixed CMP (32.9 ± 8.6%) was comparable to patients with ICM (32.7 ± 8.3%; *p* = 0.8) and lower compared to patients with NICM (40.9 ± 14.2%; *p* < 0.001). The proportion of male gender was 82% in mixed CMP, 92% in ICM and 66.7% in NICM. Patients with mixed CMP, in comparison with ICM, had lesser proportions of diabetes mellitus (33.5% vs 44.8%; *p* = 0.03), higher proportions of alcohol abuse (22.4% vs 8%; *p* < 0.001) and malignancy (30.4% vs 2.7%; *p* < 0.001), and comparable proportions of hypertension, chronic lung diseases and chronic kidney diseases (Table 1; Supplemental Table 1). Patients with mixed CMP, in comparison with NICM, had higher proportions of diabetes mellitus (33.5% vs 13.5%; *p* < 0.001), systemic hypertension (62.1% vs 36.2%; *p* < 0.001), chronic lung disease (13% vs 2.1%; *p*  < 0.001), chronic kidney disease (22.4% vs 7.1%; *p* < 0.001), malignancy (30.4% vs 11.3%; *p* < 0.001) and comparable proportions of alcohol abuse (22.4% vs 17%; *p* = 0.2).

The distribution of moderate CAD in patients with mixed CMP was LM/LAD (22.4%), LCX/RCA (1.8%), double vessel disease (56.6%) and triple vessel disease (18.6%). The level of agreement was strong (kappa = 0.81 for grading of stenosis and 0.83 for location of CAD) between the two cardiologists. The coexisting nonischemic aetiologies in the patients of mixed CMP were post myocarditis sequelae (32.9%), chemotherapy-related (24.2%), tachycardiomyopathy (19.3%), alcohol-related (16.1%) and hypertrophic cardiomyopathy (7.5%). The nonischemic aetiologies in the patients of NICM were idiopathic (23%), ARVC (11%), restrictive CMP (22.7%), valvular heart diseases (12.8%), inflammatory (10.6%), chemotherapy-related (5%), tachycardiomyopathy (6.4%), alcohol-related (7.1%) and congenital heart diseases (1.4%).

The proportion of patients receiving ICD for secondary prevention in mixed CMP was 44.1% compared to 56.3% in ICM (*p* = 0.02) and 38.3% in NICM (*p* = 0.3). While history of sudden cardiac arrest was comparable amongst all the 3 groups (23.6% in mixed CMP, 20.5% in ICM and 16.3% in NICM), incidence of atrial fibrillation was higher in mixed CMP (55.3%) compared to ICM (28.6%; *p* < 0.001) and NICM (30.5%; *p* < 0.001). While usage of beta blockers was comparable amongst all the 3 groups (> 95%), amiodarone usage was highest in ICM (38%). With respect to the distribution of type of ICD implant, patients with mixed CMP had higher proportions of CRT-d (29.2%) compared to patients with ICM (18.4%; *p* = 0.04) and NICM (18.4%; *p* = 0.03).

### Analysis of device therapies (Table 1; Supplemental Table 2)

The proportion of patients with mixed CMP receiving device therapies (34.2%) and device shocks (23.6%) was intermediate between ICM (device therapies 41.1%; device shocks 27.2%) and NICM (device therapies 29.1%; device shocks 18.4%). These differences were not significant between mixed CMP and the other groups. Among the patients receiving device shocks, the distribution of appropriate and inappropriate shocks was comparable between all the 3 groups. The minimum VT cycle length recorded in patients with mixed CMP (281.6 ± 43.1 ms) was comparable to that in ICM (282.5 ± 44 ms; *p* = 0.9) in ICM and lesser than in NICM (297.7 ± 48.7 ms; *p* = 0.1).

#### Survival characteristics

**Fig. 2:**
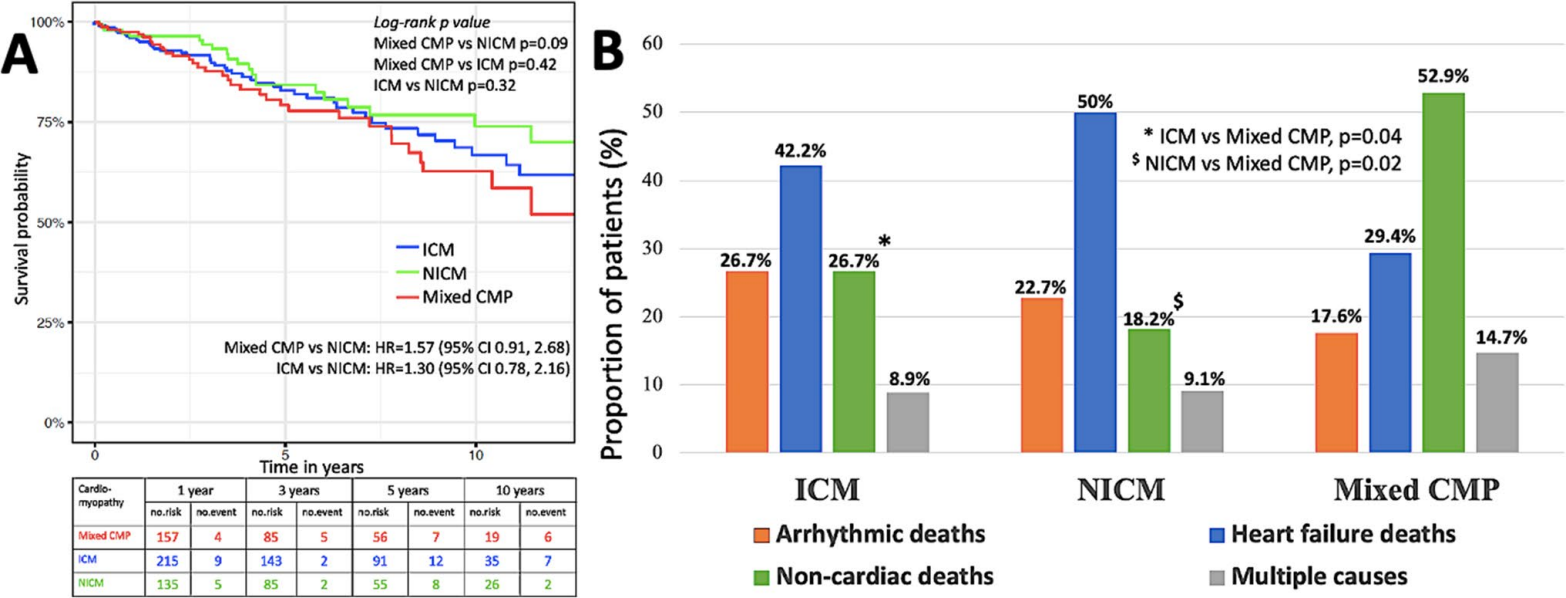
**A** Shows the Kaplan–Meier curves of survival in the three groups of cardiomyopathies with event rates at different time intervals; **B** shows the distribution of cause of deaths in the three groups of cardiomyopathies

The all-cause mortality in patients in mixed CMP (21.1%) was similar to that observed in ICM (20.1%; *p* = 0.8) and higher than in NICM (15.6%; *p* = 0.2). Time-adjusted survival estimated using the Kaplan-Meir curves revealed hazards of 1.57 (95% CI: 0.91, 2.68; *p* = 0.1) for mixed CMP compared to NICM (Fig. 2). The mean age at death in patients with mixed CMP (79 ± 8 years) was significantly higher than in ICM (73 ± 12 years; *p* = 0.01) and NICM (66 ± 14 years; *p* < 0.001). Analysis of the cause of death revealed higher proportion of non-cardiac deaths in patients with mixed CMP (52.9%), compared to ICM (26.7%; *p* = 0.04) and NICM (18.2%; *p* = 0.02). The distribution of heart failure related deaths and sudden cardiac deaths was similar between all the 3 groups (Table 2).

**Table 2:** Mortality characteristics in the three groups of cardiomyopathies in patients with ICD implant

Variables	Total (*n* = 101)	ICM (*n* = 224)	NICM (*n* = 141)	Mixed CMP (*n* = 161)
Age at death (years)	70 ± 13	73 ± 12	66 ± 14	79 ± 8^+, $^
Time-to-death (years)	5.2 ± 3.9	5.4 ± 4	5.4 ± 4.1	4.7 ± 3.5
All-cause mortality	101 (19.2)	45 (20.1)	22 (15.6)	34 (21.1)
Cardiac deaths	53 (52.5)	28 (62.2)	14 (63.6)	11 (32.4)^+, $^
Noncardiac deaths	34 (33.7)	12 (26.7)	4 (18.2)	18 (52.9)^+, $^
Multiple causes	11 (10.9)	4 (8.9)	2 (9.1)	5 (14.7)
Unknown deaths	3 (3)	1 (2.2)	2 (9.1)	0 (0)
Heart failure related	40 (39.6)	19 (42.2)	11 (50)	10 (29.4)
Arrhythmia related	23 (22.8)	12 (26.7)	5 (22.7)	6 (17.6)
Unknown cardiac deaths	1 (0.1)	1 (2.2)	0 (0)	0 (0)

*ICD* implantable cardioverter-defibrillator, *ICM* ischemic cardiomyopathy, *NICM* nonischemic cardiomyopathy, *Mixed CMP* mixed cardiomyopathy

*P* values < 0.05 have been denoted as + for the significant differences between mixed CMP and ICM groups, $ for the significant differences between Mixed CMP and NICM groups

Categorical variables have been presented as frequencies (proportions in %), Continuous variables have been presented as mean ± standard deviation with 95% confidence intervals

The Cox regression analysis (Table 3) revealed the following significant predictors of mortality age (HR: 1.04; 95% C.I: 1.02–1.06), LVEF (HR: 0.96; 95% C.I: 0.93–0.99), CKD (HR: 2.9; 95% C.I: 1.9–4.5), NYHA class (HR: 1.7; 95% C.I:1.1–2.4) and CAD (HR: 1.9; 95% C.I: 1.1–3.2). This model accounted for various confounding variables including age, gender, clinical variables, presence or absence of moderate–severe CAD and documented nonischemic triggers. Compared to the survivors in the whole cohort, the non-survivors had significantly (*p* < 0.05) higher mean age (69.1 ± 11.8y vs 62.7 ± 13y), lower LVEF (29.7 ± 6.6% vs 36.2 ± 11.3%), higher NYHA class III (51.5% vs 19.8%), lower GFR (65.8 ± 27.8 vs 85.5 ± 25.2), and significantly higher incidences of comorbidities—hypertension (64.4% vs 52.9%), chronic kidney disease (46.5% vs 10%), malignancy (23.8% vs 11%). The distribution of ICM, NICM and mixed CMP was similar (Supplemental Table 3). The proportion of patients receiving therapies was significantly higher in the non-survivors compared to the survivors (50% vs 32.3%, *p* = 0.001). Among the patients receiving device therapies, significantly higher proportion of patients received shocks in non-survivors compared to survivors (79.6% vs 63.7%, *p* = 0.04).

**Table 3:** Cox-regression stepwise model for significant predictors of mortality

Variables*	Hazards ratio	95% C.I (lower)	95% C. I (upper)	*P* value
Age at time of ICD implant	1.04	1.02	1.06	< 0.001
Chronic kidney disease	2.93	1.89	4.51	< 0.001
NYHA class	1.65	1.13	2.41	0.01
Coronary artery disease ^$^	1.88	1.11	3.17	0.02
Left ventricular ejection fraction	0.96	0.93	0.99	0.03

*ICD* implantable cardioverter-defibrillator, *NYHA* New York Heart Association classification

^*^Other nonsignificant variables in the model: male gender, diabetes mellitus, hypertension, chronic lung diseases, malignancy, alcohol abuse, history of ventricular tachycardia or sudden cardiac arrest and documented nonischemic triggers

^$^Coronary artery disease represents angiography-detected epicardial stenosis ≥ 50%

## Discussion

The salient features of our study are as follows: (1) the phenotype of mixed CMP, when compared to NICM, is associated with higher mean age and higher incidence of comorbidities; (2) ventricular arrhythmias in mixed CMP resembles ICM in terms of number of device shocks and VT cycle length; and (3) trends of long-term prognosis of patients with mixed CMP is worse than NICM and similar to ICM.

### Extent of CAD in dilated cardiomyopathy

When accounted for moderate CAD, our study reveals that at least 53% of the NICM cohort, with known nonischemic triggers, would be reclassified as mixed CMP. This cohort accounts to 30.6% of the total cohort of cardiomyopathies in our study. Cardiomyopathies with overlapping ischemic and nonischemic aetiologies are not uncommon in clinical practice [11]. In a histopathological study on hearts excised at transplantation in patients diagnosed with idiopathic DCM, coronary atherosclerosis was diagnosed in 65.5% of the hearts with 43.6% showing moderate to severe lesions [13].

In our study, nearly 77% of the mixed CMP patients had moderate CAD in more than one epicardial vessel and the majority had double vessel involvement. Concomitant CAD in DCM has been studied previously; however, they have been largely on idiopathic DCM. In addition, the results of prognosis reported in these studies are contradictory. In a study on idiopathic DCM patients, CAD burden had significant correlation with major adverse cardiovascular events [8]. Yet another large-scale study in over 12,000 heart failure patients had also shown that the prognosis in nonobstructive CAD (< 70% stenosis) is worse than in heart failure with no CAD [9]. However, a few other studies did not show differences in survival between idiopathic DCM with moderate CAD and no CAD [4, 7]. Our study is different from the above studies in that it reveals poor prognosis in patients with implanted defibrillators and CMP secondary to definite nonischemic triggers and with concomitant CAD ($\ge$ 50% to < 75% stenosis). This subset has been largely excluded from the previous studies of DCM with coexisting CAD.

### The phenotype of mixed CMP

We found mixed CMP more common in the elderly and male patients when compared to both ICM and NICM. Also, the clinical phenotype in mixed CMP seems to represent a subset of patients with higher incidences of comorbidities, especially hypertension, chronic kidney diseases, atrial fibrillation and malignancies, when compared to NICM. It is perceivable that these risk factors would also explain a relatively higher burden of CAD found in the group with mixed CMP compared to NICM [14, 15]. This finding is also consistent with the studies on idiopathic DCM with coexisting CAD [7-9]. While the proportion of device therapies and device shocks in mixed CMP falls in an intermediate category between ICM and NICM, the recorded minimum VT cycle length is comparable to patients with ICM. In a very recent study, albeit in a small cohort of 24 patients with mixed CMP undergoing catheter ablation for ventricular arrhythmias, it was shown that this subset had a higher incidence of ventricular arrhythmias and all-cause mortality than both ICM and NICM [10]. Our study reveals all-cause mortality rates of nearly 20% in both the ICM and mixed CMP cohorts. As the mean age and incidences of coexisting illnesses especially chronic kidney diseases and malignancies are higher in the cohort of mixed CMP, it is not surprising that most of the deaths in this cohort are non-cardiac, unlike the predominantly cardiac deaths in ICM and NICM. The mixed CMP group revealed higher hazards of all-cause mortality when compared to NICM (HR: 1.57; 95% CI: 0.91–2.68; *p* = 0.1). In a larger study of 2254 heart failure patients with nonobstructive CAD, when compared to 2656 heart failure patients with no CAD, there was an increased hazard of cardiovascular death (HR: 1.82; 95% CI: 1.27–2.62; *p* < 0.001) and all-cause mortality (HR: 1.18; 95% CI: 1.05–1.33; *p* < 0.005) [9].

### Possible pathogenesis in mixed CMP (Central Illustration- Fig. 3)

**Fig. 3:**
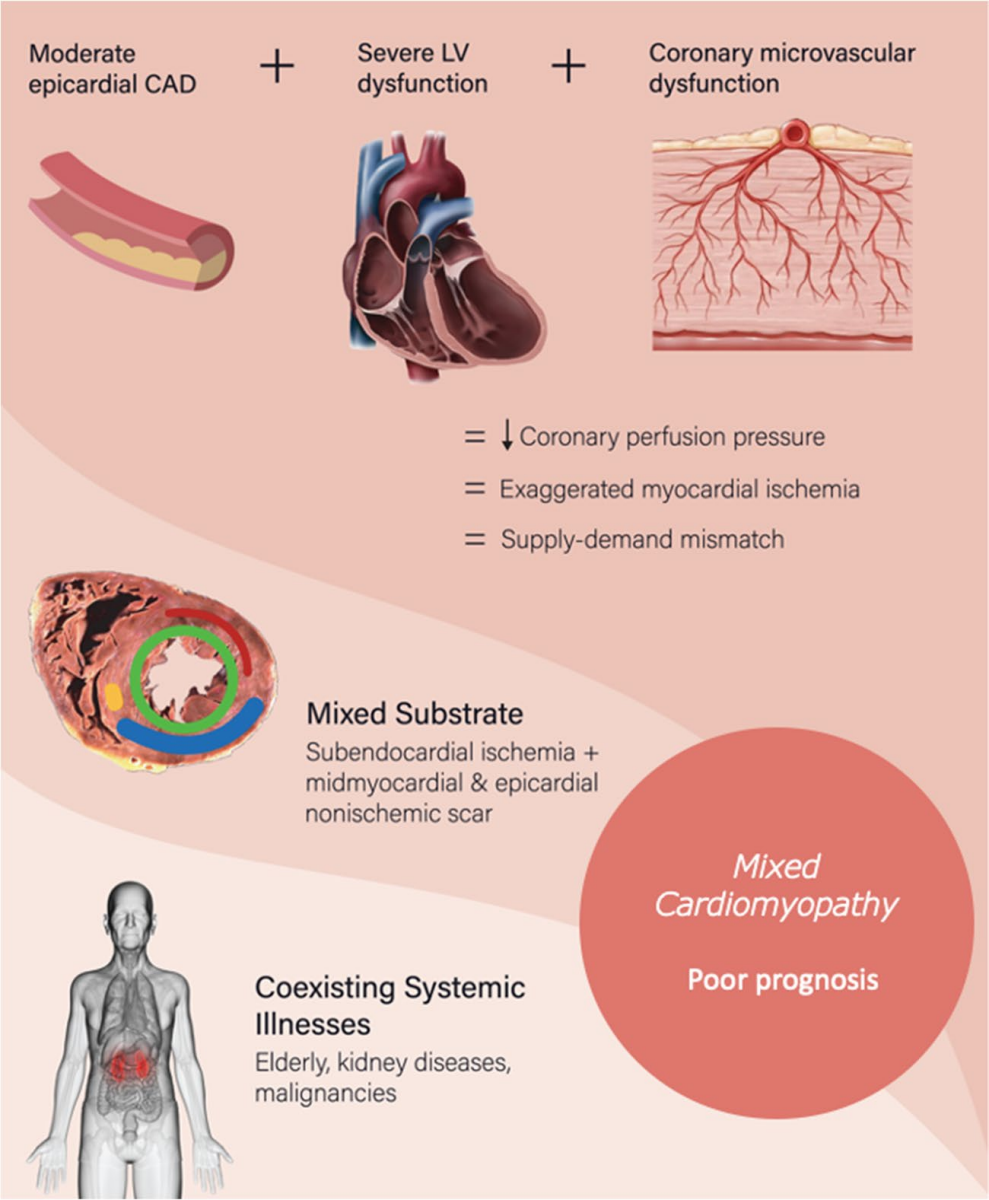
Central illustration summarises the key findings and elaborates the possible pathogenesis in Mixed Cardiomyopathy

While epicardial CAD is only one determinant of myocardial ischemia, there are multiple contributing factors: (1) supply–demand mismatch due to the low coronary perfusion pressures in the setting of severe myocardial dysfunction, (2) coronary microvascular dysfunction secondary to atherosclerosis, (3) impaired myocardial metabolic control due to the underlying CMP [16]. Coronary perfusion indices like flow reserves and microvascular resistance have been shown to be associated with poor prognosis in heart failure independent of ischemic or nonischemic classification [6, 17, 18]. Electro anatomical mapping studies have highlighted the mixed pathophysiological substrate in this subset of mixed CMP [10, 19, 20]. Such mixed pathological substrates have also been documented in small-scale studies with LGE-CMRi as well as with perfusion-CMRi [8, 21, 22]. While these can be plausible explanations for the bad prognosis in mixed CMP, there could be several other contributing factors as well like age and coexisting illnesses.

### Limitations

This is a retrospective study focussing on characterising the phenotype of mixed CMP, and hence the causal relationship between moderate CAD and depressed systolic function could not be sought. Whether or not myocardial revascularisation would benefit these patients in the presence of a demonstrable myocardial ischemia, has to be explored prospectively. Also, scoring of the extent of CAD and its burden with indices or variables like focal or diffuse involvement and location is likely to throw more light into the incremental effect of each variable on the perfusion abnormality [4, 8]. A larger sample size could have established statistical significance to the observed higher trends of mortality in mixed CMP compared to NICM. Finally, though this is the first study to address the phenotype of mixed CMP in patients implanted with defibrillators, and hence arbitrary definitions were employed for the categorisation of mixed CMP.

## Conclusion

Our study characterises the mixed phenotype of dilated cardiomyopathies who have established nonischemic triggers and concomitant moderate CAD, in a cohort who had received an ICD. The prognosis in patients with mixed CMP, with regards to device therapies and all-cause mortality, resembles ICM. The prognosis in patients with mixed CMP is poorer than NICM in terms of significantly higher burden of comorbidities, poorer LV functions and trend towards higher proportions of device shocks and higher mortality. The higher mortality seems to be driven by higher incidences of non-cardiac deaths, thus representing a sicker subset than NICM. Large-scale studies focusing on this phenotype need to assess the mediators of poorer prognosis due to the underlying pathophysiological substrate and the associated coexisting illnesses.

### Supplementary Information

Below is the link to the electronic supplementary material.

Supplementary file1 (DOCX 40 KB)

## Data Availability

The data that support the findings of the study are available on request from the corresponding author.

## Abbreviations

CAD: Coronary artery disease
CAG: Coronary angiogram
CKD: Chronic kidney disease
CMP: Cardiomyopathy
CMRi: Cardiac magnetic resonance imaging
DCM: Dilated cardiomyopathy
EGM: Electrogram
HR: Hazard ratio
ICD: Implantable cardioverter-defibrillator
ICM: Ischemic cardiomyopathy
LAD: Left anterior descending artery
LCX: Left circumflex artery
LGE: Late gadolinium enhacement
LV: Left ventricle
LVEF: Left ventricular ejection fraction
MI: Myocardial infarction
MRI: Magnetic resonance imaging
NICM: Nonischemic cardiomyopathy
NYHA: New York heart association
NDI: National death index
PCI: Percutaneous coronary intervention
PET: Positron emission tomography
RCA: Right coronary artery
SCD: Sudden cardiac death
SCA: Sudden cardiac arrest
VT: Ventricular tachyarrhythmia
