# Supplementary material for: Characteristics of the phenotype of mixed cardiomyopathy in patients with implantable cardioverter-defibrillators
Source: J Interv Card Electrophysiol. 2023 Jun 5;67(1):129–37. doi: 10.1007/s10840-023-01577-x (PMC10770238; doi:10.1007/s10840-023-01577-x)
Supplement: Supplementary file 1 — Supplementary file1 (DOCX 40 KB) [file 10840_2023_1577_MOESM1_ESM.docx]

**Supplementary material**

**Inclusion criteria:**

1. All patients who received an ICD anytime from January 01, 2005 until June 30, 2019 at the Canberra hospital.
2. Patients who had atleast one interrogation (clinical or remote transmission) of the implanted ICD in the follow-up.
3. Patients who had an invasive coronary angiogram to rule out coronary artery disease.

**Exclusion criteria:**

1. Those patients with not even one interrogation of the implanted ICD in the follow-up were excluded for analysis of device therapies.
2. Those patients with an in-hospital or 30-day mortality after the procedure were excluded for analysis of survival characteristics.
3. Those patients with incomplete hospital records limiting collection of any meaningful clinical data.

**Data collection:**

1. Name, age, gender, treating physician, history of diabetes, hypertension, chronic kidney disease (CKD), lung disease, malignancy, alcohol/ drug abuse.
2. Kidney functions namely serum creatine and estimated glomerular filtration rate (eGFR) and echocardiographic findings including type and severity of valve pathologies, left ventricular ejection fraction (LVEF) at implant were recorded. LVEF at the last follow-up was recorded.
3. History of coronary artery disease (CAD)/ myocardial infarction (MI)/ type of MI- ST elevation (STEMI) and non-ST elevation (NSTEMI)/ percutaneous coronary intervention (PCI)/ history of bypass surgery/ valve replacement/ major non-vascular surgeries.
4. Documented arrhythmias- supraventricular (inclusive of atrial flutter and fibrillation) and ventricular (shockable rhythms like ventricular fibrillation, monomorphic and polymorphic ventricular tachycardias).
5. List of anti-arrhythmic and heart failure medications.
6. Symptoms of syncope or sudden cardiac arrest (SCA) or documented VT.
7. Categorization of patients into inherited channelopathies or cardiomyopathies were done as per study definitions.
8. History of radiofrequency ablation (RFA) for SVT and VT in relation to the time of ICD implant.
9. Device characteristics: information on clinical interrogation during a scheduled clinic visit or remote transmission, type and manufacturer of ICD, complications during implant, the programming zones of the ICD, date of first and second therapy from the device, verification of the type of tachyarrhythmia and the type of therapies delivered verified with the stored intracardiac electrograms (EGMs), change in the programming parameters, minimum cycle length of the recorded ventricular tachyarrhythmia (both 1^st^ and 2^nd^ episode), VT storms, date and number of generator changes, therapies after generator change.
10. Survival characteristics: Survival data as of end of June 2020, cause and date of death.

**Study definitions:**

1. Syncope was defined as a witnessed episode of loss of consciousness associated with loss of postural tone with spontaneous recovery.
2. Sudden cardiac arrest (SCA) was defined as out-of-hospital events of successful cardio-pulmonary resuscitation with or without direct current shocks delivered by an external cardioverter-defibrillator.
3. Non-sustained ventricular tachycardia (NSVT) was defined as ≥ 3 consecutive ventricular premature beats with a rate >100 beats/min, lasting < 30s and without hemodynamic instability. NSVT should have been documented during exercise testing, loop monitoring or 24-h Holter monitoring.
4. Diabetes mellitus and hypertension were considered if the patient was on long standing medications for treatment of these conditions.
5. Chronic kidney disease was diagnosed if the patients were already categorised so by the treating physician based on evidence of kidney damage like elevated serum creatine or reduced estimated glomerular filtration rate (<60ml/mt/1.73sq.mt) with or without ongoing haemodialysis.
6. Chronic lung disease was diagnosed if the patients were already categorised so by the treating physician based on evidence of suggestive clinical symptoms requiring treatment for the same.
7. Malignancy was diagnosed if the patients were already categorised so by the treating physician based on evidence of clinical symptoms, biochemical markers or imaging evidence requiring one or more of chemotherapy or radiation therapy or surgical removal. The organ involved was also noted.
8. Coronary artery disease (CAD) was defined by the presence of stenosis $\geq$50% in atleast one of three major epicardial vessels or $\geq$30% in the left main vessel. Lesions on coronary angiography (CAG) were graded visually by two cardiologists on the following ordinal scale: 0% to <50%, $\geq$ 50% to <75%, $\geq$ 75% and 100%. The interobserver agreement for both grading of stenosis and location of CAD was calculated. The final consensus was reached upon by mutual agreement.
9. Primary prevention of SCD referred to use of ICDs in individuals who are at risk for but have not yet had an episode of sustained VT, VF or cardiac arrest. Patients with inducible VT on electrophysiology studies, but with no documented evidence of prior VT were also categorised under primary prevention.
10. Secondary prevention referred to an indication for an ICD exclusively for patients who have survived one or more cardiac arrests or sustained ventricular tachycardia.
11. Device therapies were recorded as events of either shock or anti-tachycardia pacing (ATP) delivered by the device. Number of patients receiving therapies, shocks, appropriate and inappropriate shocks, and number of each therapy per patient were noted.
12. Appropriate ICD therapy was defined as an intervention (either shock or antitachycardia pacing) triggered by a ventricular arrhythmia classified as a true event by two separate cardiac electrophysiologists.
13. Appropriate ICD shock was defined as an ICD shock triggered by a sustained ventricular arrhythmia.
14. Inappropriate ICD shock was defined as ICD shock triggered by non-sustained ventricular arrhythmias, supraventricular arrhythmias, sinus tachycardia, oversensing, or device malfunction (such as lead fracture leading to inappropriate shocks).
15. VT storm was used to denote 3 or more separate therapies either in form of ATP or shock delivered by the device in a 24-hour interval.
16. Device-related complications were grouped under pocket revisions due to local infection or hematoma, lead revisions including lead extractions and reimplantation due to pacemaker-related infection or lead malfunctions (dislodgement, fracture, insulation break, cardiac perforation) or lead repositioning, and generator replacements due to malfunctions.
17. Follow-up duration was calculated from time of implant to the latest date of data interrogation.
18. Time to therapy was calculated from time of implant to first device therapy.
19. Time to death was calculated from time of implant to death.
20. Time to generator change was calculated from time of implant to time of first generator change.
21. Comorbidity index was used as a composite scale ranging from 1 to 5 inclusive of diabetes mellitus, hypertension, chronic kidney disease, chronic lung disease and malignancy
22. All-cause mortality was recorded as death due to any cause retrieved from the National Death Index (NDI). Cardiac and noncardiac deaths were defined respectively based on the recorded causes of death in the NDI. Heart failure and arrhythmia-related deaths were the only causes of the recorded cardiac deaths.

**Supplementary tables**

**Supplementary table 1: Clinical characteristics in the three groups of cardiomyopathies in patients with ICD implant**

| Variables | Total (n=526) | ICM (n=224)  Group A | NICM (n=141)  Group B | Mixed CMP (n=161)  Group C | P value  A vs B | P value  A vs C | P value  B vs C |
| --- | --- | --- | --- | --- | --- | --- | --- |
| Age (years) | 64±13 | 66.3±10.9 | 54.4±14.5 | 69.1±9.6 | <0.001 | 0.008 | <0.001 |
| Male | 432 (82.1) | 206 (92) | 94 (66.7) | 132 (82) | <0.001 | 0.004 | 0.003 |
| Diabetes mellitus | 173 (33) | 100 (44.8) | 19 (13.5) | 54 (33.5) | <0.001 | 0.03 | <0.001 |
| Hypertension | 290 (55.1) | 139 (62.1) | 51 (36.2) | 100 (62.1) | <0.001 | 1 | <0.001 |
| Chronic lung diseases | 45 (8.6) | 21 (9.4) | 3 (2.1) | 21 (13) | 0.01 | 0.3 | <0.001 |
| CKD | 91 (17.3) | 45 (20.1) | 10 (7.1) | 36 (22.4) | 0.001 | 0.6 | <0.001 |
| Alcohol abuse | 60 (11.4) | 0 (0) | 24 (17) | 36 (22.4) | <0.001 | <0.001 | 0.2 |
| Malignancy | 71 (13.5) | 6 (2.7) | 16 (11.3) | 49 (30.4) | 0.001 | <0.001 | <0.001 |
| Atrial fibrillation | 196 (37.3) | 64 (28.6) | 43 (30.5) | 89 (55.3) | 0.7 | <0.001 | <0.001 |
| LVEF at baseline | 35±10.9 | 32.7±8.3 | 40.9±14.2 | 32.9±8.6 | <0.001 | 0.8 | <0.001 |
| Creatine | 94.5±45.9 | 101.5±54 | 84.6±37.5 | 93.1±38 | 0.002 | 0.09 | 0.06 |
| GFR | 81.7±26.8 | 79±29 | 89±25.7 | 79.2±23.4 | 0.001 | 0.9 | 0.001 |
| CAD | 385 (73.2) | 224 (100) | 141 (100) | 161 (100) | <0.001 | - | <0.001 |
| MI | 216 (41) | 216 (96) | 0 (0) | 0 (0) | - | - | - |
| PCI | 117 (30.7) | 117 (53.2) | 0 (0) | 0 (0) | <0.001 | <0.001 | - |
| CABG | 100 (26) | 100 (44.8) | 0 (0) | 0 (0) | <0.001 | <0.001 | - |
| Primary prevention | 270 (52.3) | 98 (43.8) | 87 (61.7) | 90 (55.1) | 0.001 | 0.02 | 0.3 |
| Secondary prevention | 251 (47.7) | 126 (56.3) | 54 (38.3) | 71 (44.1) |  |  |  |
| NYHA class 2 | 282 (53.6) | 150 (67) | 56 (39.7) | 76 (47.2) | <0.001 | 0.001 | 0.001 |
| NYHA class 3 | 136 (25.9) | 50 (22.3) | 33 (23.4) | 53 (32.9) |  |  |  |
| NYHA class 4 | 4 (0.8) | 1 (0.4) | 0 (0) | 3 (1.9) |  |  |  |
| History of syncope | 119 (22.6) | 39 (17.4) | 45 (31.9) | 35 (21.7) | 0.02 | 0.5 | 0.2 |
| Cardiac arrest | 107 (20.3) | 46 (20.5) | 23 (16.3) | 38 (23.6) | 0.3 | 0.5 | 0.2 |
| Documented VT | 165 (31.4) | 88 (39.3) | 34 (24.1) | 43 (26.7) | 0.002 | 0.002 | 0.4 |
| Betablocker usage | 504 (96.4) | 215 (96) | 133 (95.7) | 156 (97.5) | 0.9 | 0.5 | 0.5 |
| Amiodarone usage | 169 (32.3) | 85 (37.9) | 39 (28.1) | 45 (28.1) | 0.07 | 0.05 | 1 |
| ACEi-ARB usage | 383 (73.2) | 174 (77.7) | 78 (56.1) | 131 (81.9) | <0.001 | 0.3 | <0.001 |
| VT ablation | 56 (10.6) | 27 (12.1) | 16 (11.3) | 13 (8.1) | 0.9 | 0.2 | 0.4 |
| Categorical variables are expressed in number (proportion in %); Continuous variables are expressed as mean ±SD with 95% confidence intervals; ICD- Implantable Cardioverter-Defibrillator; ICM- ischaemic cardiomyopathy; NICM- nonischaemic cardiomyopathy; Mixed CMP- mixed cardiomyopathy; CKD- chronic kidney disease; LVEF- left ventricular ejection fraction; CAD- coronary artery disease; MI- myocardial infarction; PCI- percutaneous coronary intervention; CABG- coronary artery bypass surgery; NYHA- New York heart association; VT- ventricular tachycardia; ACEi- angiotensin converting enzyme inhibitor; ARB- angiotensin receptor blocker | | | | | | | |

**Supplementary table 2: Device therapy characteristics in the three groups of cardiomyopathies in patients with ICD implant**

| **Variables** | **Total (n=526)** | **ICM**  **(n=224)**  **Group A** | **NICM (n=141)**  **Group B** | **Mixed CMP (n=161)**  **Group C** | **P value**  **A vs B** | **P value**  **A vs C** | **P value**  **B vs C** |
| --- | --- | --- | --- | --- | --- | --- | --- |
| Single chamber | 196 (37.3) | 81 (36.2) | 59(41.8) | 56 (34.8) | 0.04 | 0.3 | 0.06 |
| Dual chamber | 190 (36.1) | 90 (40.2) | 46 (32.6) | 54 (33.5) |  |  |  |
| CRT | 121 (23) | 48 (21.4) | 26 (18.4) | 47 (29.2) | 0.5 | 0.09 | 0.03 |
| Remote monitoring | 194 (36.9) | 70 (31.3) | 59 (41.8) | 65 (40.4) | 0.04 | 0.06 | 0.8 |
| Pacemaker type 1 complications | 9 (1.7) | 5 (2.2) | 1 (0.7) | 3 (1.8) | 0.2 | 0.2 | 0.3 |
| Pacemaker type 2 complications | 23 (4.3) | 9 (4.1) | 8 (5.6) | 6 (3.7) |  |  |  |
| Pacemaker type 3 complications | 6 (1.1) | 5 (2.2) | 1 (0.7) | 0 (0) |  |  |  |
| Min VT Cycle length (millisec) | 286.8±45.6 | 282.5±44 | 297.7±48.7 | 281.6±43.1 | 0.1 | 0.9 | 0.09 |
| Therapies | 184 (35.7) | 88 (41.1) | 41 (29.1) | 55 (34.2) | 0.04 | 0.3 | 0.3 |
| Shocks received | 125 (23.7) | 61 (27.2) | 26 (18.4) | 38 (23.6) | 0.05 | 0.3 | 0.4 |
| Appropriate shocks received | 86 (16.3) | 42 (18.8) | 17 (12.1) | 27 (16.7) | 0.09 | 0.6 | 0.2 |
| Inappropriate shocks received | 39 (7.4) | 19 (8.4) | 9 (6.4) | 11 (6.8) | 0.5 | 0.6 | 0.9 |
| No. of therapies | 5 (2, 15.8) | 4.5 (1, 12) | 4 (2, 16) | 8 (2, 27) | 0.3 | 0.5 | 0.2 |
| No. of shocks | 3 (1, 8.5) | 3 (1, 9) | 4 (2, 7) | 3 (2, 8.5) | 0.3 | 0.4 | 0.6 |
| No. of approp. shocks | 4 (2, 9) | 4 (1, 9) | 5 (3, 16) | 3 (2, 8) | 0.8 | 0.8 | 0.1 |
| No. of inapprop. shocks | 2 (1, 3) | 1 (1, 3) | 2 (1, 2) | 3.5 (1, 6.5) | 0.8 | 0.2 | 0.1 |
| VT storms | 44 (8.3) | 18 (8) | 14 (9.9) | 12 (7.4) | 0.9 | 0.9 | 0.7 |
| No. of VT storms | 1 (1, 2) | 1 (1, 2.3) | 1 (1, 2.3) | 1.5 (1, 2) | 0.9 | 0.7 | 0.6 |
| No. of AADs | 1 (1, 2) | 1 (1, 2) | 1 (1, 1) | 1 (1, 1) | 0.01 | 0.01 | 0.8 |
| Box change | 96 (18.3) | 40 (17.9) | 31 (22) | 25 (15.5) | 0.3 | 0.6 | 0.2 |
| No. of box changes | 1 (1, 1) | 1 (1, 1) | 1 (1, 1) | 1 (1, 1) | 0.9 | 0.8 | 0.8 |
| Therapies post box change | 34 (34.7) | 16 (39) | 9 (29) | 9 (34.6) | 0.5 | 0.8 | 0.7 |
| Time to device therapy (years) | 2.4±2.8 | 2.4±2.7 | 2.8±3.4 | 2±2.3 | 0.5 | 0.5 | 0.3 |
| Time to approp. shock (years) | 2.3±2.8 | 2.4±2.9 | 2.6±3.5 | 1.8±1.9 | 0.7 | 0.2 | 0.2 |
| Categorical variables are expressed in number (proportion in %); Continuous variables are expressed as mean ±SD with 95% confidence intervals; No. are presented in medians (25^th^, 70^th^ percentiles)  ICD- Implantable Cardioverter-Defibrillator; CRT- Cardiac resynchronisation therapy; AAD- antiarrhythmic drugs; Type 1 complications- pocket infection/ erosion/ hematoma/ device extraction; Type 2 complications- lead revision/ fracture/ perforation; Type 3 complications- generator malfunction; VT- ventricular tachycardia; No- number; Approp.- appropriate; Inapprop.- inappropriate | | | | | | | |

**Supplementary table 3:** Clinical and device therapy characteristics in survivors and nonsurvivors in patients with ICD implant

| **Variables** | **Survivors** | **Nonsurvivors** | **P value** | **Variables** | **Survivors** | **Nonsurvivors** | **P value** |
| --- | --- | --- | --- | --- | --- | --- | --- |
| Age (years) | 62.7±13 | 69.1±11.8 | <0.001 | Single chamber | 153 (36) | 43 (42.6) | 0.2 |
| Male | 345 (81.2) | 87 (86.1) | 0.3 | Dual chamber | 154 (36.2) | 36 (35.6) |  |
| Diabetes | 136 (32.1) | 37 (36.6) | 0.4 | CRT | 99 (23.3) | 22 (21.8) | 0.8 |
| Hypertension | 225 (52.9) | 65 (64.4) | 0.04 | Pacemaker type I complications | 8 (22.2) | 1 (50) | 0.6 |
| COPD | 29 (6.8) | 16 (15.8) | 0.005 | Pacemaker type 2 complications | 22 (61.1) | 1 (50) |  |
| CKD | 44 (10) | 47 (46.5) | <0.001 | Pacemaker type 3 complications | 6 (16.7) | 0 (0) |  |
| Alcohol abuse | 57 (13.4) | 3 (3) | 0.01 | Therapies received | 135 (32.3) | 49 (50) | 0.001 |
| Malignancy | 47 (11.1) | 24 (23.8) | 0.003 | Shocks received | 86 (63.7) | 39 (79.6) | 0.04 |
| AF | 152 (35.8) | 44 (43.6) | 0.2 | Appropriate shocks received | 55 (66.3) | 30 (83.3) | 0.08 |
| LVEF | 36.2±11.3 | 29.7±6.6 | <0.001 | Inappropriate shocks received | 28 (33.7) | 6 (16.7) | 0.07 |
| GFR | 85.5±25.2 | 65.8±27.8 | <0.001 | VT storms | 26 (6) | 18 (17.8) | <0.001 |
| Cardiac arrest | 84 (19.8) | 23 (22.8) | 0.5 | Min VT CL-ms | 292.5±46 | 269.4±38 | 0.007 |
| Syncope | 94 (22.1) | 25 (24.8) | 0.7 | Box change | 70 (17.6) | 21 (20.8) | 0.5 |
| Documented VT | 124 (29.2) | 41 (40.6) | 0.1 | Therapies post box change | 22 (28.9) | 12 (54.5) | 0.04 |
| ICM | 179 (42.1) | 45 (44.6) | 0.6 | No. of therapies | 4 (2, 13) | 6 (2, 25.5) | 0.2 |
| NICM | 119 (28) | 22 (21.8) | 0.2 | No. of shocks | 3 (1, 9) | 3 (2, 8) | 0.9 |
| Mixed CMP | 127 (29.9) | 34 (33.7) | 0.4 | No. of approp. shocks | 3 (1, 10) | 4.5 (2, 9) | 0.5 |
| CAD | 306 (72) | 79 (78.2) | 0.2 | No. of inapprop. shocks | 2 (1, 4.5) | 1 (1, 3) | 0.8 |
| PCI | 97 (31.9) | 20 (26) | 0.3 | No of VT storms | 1 (1, 3) | 1 (1, 2) | 0.8 |
| CABG | 80 (26.1) | 20 (25.6) | 0.9 | No of box changes | 1 (1, 1) | 1 (1, 1) | 0.5 |
| Noncardiac surgeries | 10 (2.4) | 6 (5.9) | 0.09 | Time to first approp. shock | 2.3±3 | 2.3±2.5 | 0.9 |
| Secondary prevention | 193 (45.4) | 58 (57.4) | 0.03 | No of AADs | 1 (1, 2) | 1 (1, 2) | 0.1 |
| NYHA II | 242 (56.9) | 40 (39.6) | <0.001 | Betablocker usage | 405 (95.7) | 99 (99) | 0.2 |
| NYHA III/IV | 86 (20.3) | 54 (53.5) |  | ACEi-ARB usage | 308 (72.8) | 70 (70) | 0.7 |
| Categorical variables are expressed in number (proportion in %); Continuous variables are expressed as mean ±SD with 95% confidence intervals; No. are presented in medians (25^th^, 70^th^ percentiles)  ICD- Implantable Cardioverter-Defibrillator; COPD- Chronic obstructive pulmonary disease; CKD- chronic kidney disease; AF- atrial fibrillation; LVEF- left ventricular ejection fraction; GFR- estimated glomerular filtration rate; VT- ventricular tachycardia; ICM- ischaemic cardiomyopathy; NICM- nonischaemic cardiomyopathy; Mixed CMP- mixed cardiomyopathy; CAD- coronary artery disease; PCI- percutaneous coronary intervention; CABG- coronary artery bypass surgery; NYHA- New York heart association; CRT- cardiac resychronisation therapy; Type 1 complications- pocket infection/ erosion/ hematoma/ device extraction; Type 2 complications- lead revision/ fracture/ perforation; Type 3 complications- generator malfunction; CL- cycle length in milliseconds (ms); No.- number; Approp.- appropriate; Inapprop.- inappropriate; ACEi- angiotensin converting enzyme inhibitor; ARB- angiotensin receptor blocker; AAD- antiarrhythmic drugs | | | | | | | |
